# Combining AKT inhibition with chloroquine and gefitinib prevents compensatory autophagy and induces cell death in EGFR mutated NSCLC cells

**DOI:** 10.18632/oncotarget.2017

**Published:** 2014-05-27

**Authors:** Sivan M. Bokobza, Yanyan Jiang, Anika M. Weber, Aoife M. Devery, Anderson J. Ryan

**Affiliations:** ^1^ Gray Institute for Radiation Oncology & Biology, Department of Oncology, University of Oxford, Old Road Campus Research Building, Headington, Oxford, UK

**Keywords:** EGFR, lung cancer, AKT, Chloroquine

## Abstract

Although non-small cell lung cancer (NSCLC) patients with EGFR mutation positive (EGFR M+) tumors initially respond well to EGFR tyrosine kinase inhibitor (TKI) monotherapy, the responses are usually incomplete. In this study we show that AKT inhibition, most importantly AKT2 inhibition, synergises with EGFR TKI inhibition to increase cell killing in EGFR M+ NSCLC cells. However, our data also suggest that the synergistic pro-apoptotic effects may be stunted due to a prosurvival autophagy response induced by AKT inhibition. Consequently, inhibiting autophagy with chloroquine significantly enhanced tumor cell death induced by gefitinib and AKT inhibitors in EGFR M+ cells *in vitro,* and produced greater tumor shrinkage in EGFR M+ xenografts *in vivo*. Together, our findings suggest that adding chloroquine to EGFR and AKT inhibition has the potential to improve tumor responses in EGFR M+ NSCLC, and that selective targeting of AKT2 may provide a new treatment option in NSCLC.

## INTRODUCTION

Lung cancer is the leading form of cancer worldwide in terms of both incidence and death [1]. Non-small cell lung cancer (NSCLC), the most common type of lung cancer is of epithelial origin and accounts for 80% of all lung cancers [2]. 15-30% of NSCLC patients have tumors harboring activating mutations in the epidermal growth factor receptor (EGFR) [3, 4]. These mutations are most commonly found in never smokers and are associated with response to EGFR tyrosine kinase inhibitors (TKIs), including gefitinib and erlotinib [3, 4]. Although a high proportion of patients with EGFR mutation positive (EGFR M+) tumors initially obtain significant clinical benefits from EGFR TKIs, acquired drug resistance usually occurs within 12 months of starting treatment [5, 6]. Therefore, there is significant unmet clinical need to improve efficacy of treatment for this group of NSCLC patients.

EGFR elicits its effects by signaling through downstream kinases such as extracellular signal-regulated kinase (ERK) and AKT [7]. AKT is the primary downstream mediator of phospho-inositide 3-kinase (PI3K) signaling, a pathway central in regulating cell proliferation, survival, death, migration, and angiogenesis [8]. It is therefore no surprise that perturbations in this pathway have been implicated in tumorigenesis. There are three AKT isoforms; AKT1, AKT2, and AKT3, which are closely related, but have been shown to have distinct functions and locations [9]. AKT1, for example, has been demonstrated to inhibit the invasion and migration of breast cancer and ovarian cancer cells [10, 11], but has also been shown to increase lung tumor cell growth, migration, and metastasis [12, 13]. In contrast, while AKT2 has been shown to promote apoptosis in lung cancer cells, it can promote invasion and metastasis of breast cancer cells [13, 14].

Despite the clear functional disparity between AKT1, 2, and 3, current attempts in the clinic to inhibit the pathway target all three AKT isoforms. The leading clinical candidate is MK2206, an orally administered allosteric AKT inhibitor that has been evaluated in several clinical trials for the treatment of solid tumors. MK2206 has so far shown only moderate efficacy both alone and in combination with other drugs and has been associated with toxicities including rash, nausea, and hyperglaecemia [15, 16]. The potential opposing roles of the specific AKT isoforms, as well as the toxicity associated with pan-AKT inhibition, suggests that the development of AKT isoform specific inhibitors may be of future interest

Amongst the many processes that AKT is involved in, AKT inhibition with the use of either AKT inhibitors or siRNA, has been shown to induce autophagy in a variety of cell types [17-19]. Autophagy is a catabolic cellular process catalysed by the ubiquitin-like LC3 in which cells recycle subcellular components into membrane vacuoles known as autophagosomes [20]. Some regulators of autophagy have been shown to function as tumor suppressors by promoting protein degradation and thereby reducing cell growth. Conversely, autophagy has been shown to act as an anti-apoptotic mechanism for tumor cells under metabolic stresses such as hypoxia and growth-factor removal [21-23]. Furthermore, the use of autophagy inhibitors has been shown to promote cancer cell death in combination with various cancer therapies [17, 24]. In this study we investigate the isoform specific roles of AKT in regulating the response of EGFR M+ NSCLC cells to gefitinib, and the impact of inhibiting prosurvival autophagy induced by AKT inhibition.

## RESULTS

### MK2206 increases sensitivity of EGFR M+ NSCLC cells to gefitinib-induced growth inhibition and clonogenic cell killing

**Figure 1:**
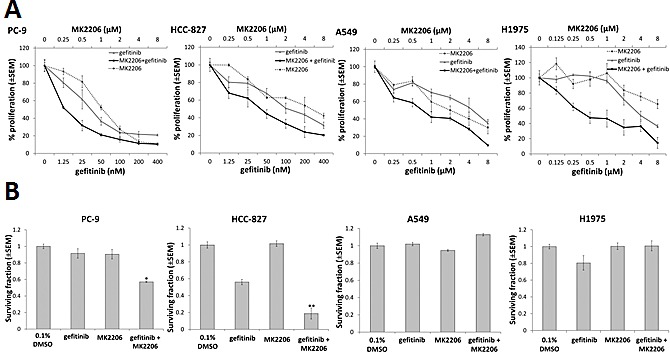
The effect of combining gefitinib and MK2206 on growth and clonogenic survival of NSCLC cells A, Proliferation assay of NSCLC cells in presence of MK2206 and/or gefitinib for 96 h. The dose range studied for each drug was approximately 0.25x to 8.0x the IC50 value for each cell line. B, Clonogenic survival of NSCLC cells treated with gefitinib (0.1μM for PC-9 and HCC-827; 1μM for A549 and H1975) and/or MK2206 (1μM) for 24 h, *P<0.05 and **P<0.01, compared to gefitinib alone, but there was no difference observed in A549 or H1975 cells. Data are mean ± SEM (n=3).

EGFR M+ NSCLC cells (HCC-827, PC-9) were markedly more sensitive to growth inhibition by an EGFR TKI (gefitinib) as well as the AKT inhibitor MK2206, compared with EGFR wild-type cells (A549) and EGFR T790M cells (H1975) (Table 1). The method of Chou and Talalay [25] was used to assess the effects of the combination of gefitinib and MK2206 on NSCLC cell proliferation. Cells were treated with gefitinib and MK2206 alone, or in combination, at a fixed concentration ratio of 1:20 (PC-9 and HCC-827) or 1:1 (A549 and H1975), according to the approximate ratios of the IC50s for each drug (Fig. 1A). The combination index (CI) value for each of the cell lines was <1 at ED50 and ED75 (Table 2), suggesting that MK2206 and gefitinib had synergistic effects on growth inhibition in both EGFR WT and EGFR M+ cell lines.

**Table 1:** IC50s of gefitinib and MK2206 for the NSCLC cell lines used in this study

Cell line	IC50 (μM ± SEM)	
	gefitinib	MK2206
PC-9	0.07±8x10-2	1.18±0.23
HCC-827	0.03±8x10-5	2.25±0.25
A549	5.25±0.77	4.81±0.43
H1975	9.57±1.94	7.66±0.63

**Table 2:** CI values for the combination of MK2206 and gefitinib in NSCLC cells

Cell line	Combination index (±SEM)		
	ED50	ED75	ED90
PC-9	0.55±0.19	0.64±0.19	0.9±0.31
HCC-827	0.74±0.15	0.82±0.14	1.05±0.12
A549	0.50±0.15	0.69±0.20	1.18±0.61
H1975	0.26±0.02	0.94±0.03	3.48±0.09

To assess clonogenic survival, cells were treated with gefitinib (0.1 μM for HCC-827 and PC-9 or 1 μM for A549 and H1975) and/or MK2206 (1 μM) for 24 h, and then cultured to form colonies ≥50 cells. Whereas MK2206 did not have a significant effect on clonogenic survival, the combination of MK2206 and gefitinib significantly reduced clonogenic survival in PC-9 (−40.7±3.4%, p=0.03) and HCC-827 cells (−37.2±3.3%, p<0.01), compared with gefitinib alone, but not in A549 or H1975 cells (Fig. 1B).

### MK2206 increases sensitivity of EGFR M+ cells to gefitinib-induced apoptosis and inhibition of downstream signaling pathways

**Figure 2:**
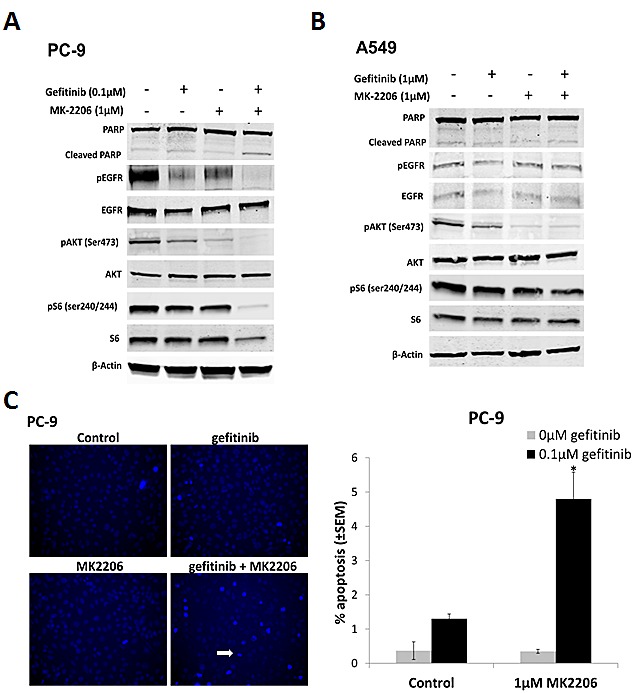
The effect of combining gefitinib and MK2206 on EGFR downstream signaling and apoptosis of NSCLC cells Western blots of A, PC-9 and B, A549 cells treated with gefitinib (0.1μM and 1μM, respectively) and MK2206 (1μM) for 24 h. The β-Actin was used as a loading control and blots are representative of at least 2 repeats. C, Drug-induced apoptosis assay. Cells were treated with gefitinib (PC-9, 0.1μM; A549, 1μM), MK2206 (1μM), or the combination, for 18 h. Cells with condensed bright nuclei (white arrow) were scored as apoptotic, *P<0.05, compared to either drug alone. Data represent mean apoptotic levels ± SEM (n=3).

To assess the effects of MK2206 and gefitinib on EGFR downstream signaling, cells were treated with either gefitinib or MK2206 alone, or in combination, for 24 h, and western blotting carried out. In PC-9 cells, treatment with gefitinib (0.1 μM) markedly reduced pEGFR and pAKT levels (Fig. 2A). Reduced pAKT levels were also evident after gefitinib treatment (1.0 μM) in A549 cells, but to a lesser extent than in PC-9 cells (Fig. 2B). MK2206 treatment (1.0 μM) on the other hand, significantly reduced the levels of pAKT in both cell lines. Combining MK2206 and gefitinib further reduced both pAKT and pEGFR levels in PC-9 cells, but not in A549 cells (Fig. 2A and B). In addition, combined treatment with gefitinib and MK2206 for 24 h in PC-9 (but not A549) cells resulted in an increase in levels of cleaved PARP compared with gefitinib or MK2206 alone, suggesting increased levels of apoptosis (Fig. 2A and B).

To assess the effects of combined drug treatment on apoptosis, cells were treated with gefitinib (PC-9; 0.1 μM, and A549; 1 μM) for 18 h, and stained with Hoechst 33258. Hoechst fluorescence was imaged in live cells, and cells with condensed nuclei and high staining intensity scored as apoptotic (Fig. 2C). Gefitinib or MK2206 treatment alone induced low levels of apoptosis in both cell lines. When PC-9 cells were treated with both drugs concurrently, the percentage of apoptotic cells was significantly increased, compared with gefitinib alone (Fig. 2C), but not in A549 cells (data not shown).

### Selective inhibition of AKT protein isoforms using siRNA augments gefitinib response in EGFR M+ cells

In order to determine relative contribution of AKT isoforms, siRNAs against AKT1, 2, and 3, and total AKT, respectively, were introduced into PC-9 and A549 cells and efficient protein knock-down confirmed by western blotting (Supplementary Fig. S1).

**Figure 3:**
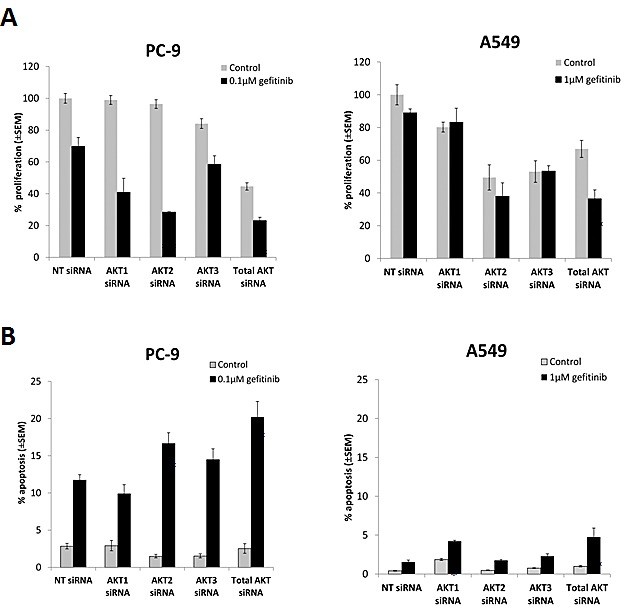
The effect of selective inhibition of AKT isoforms with siRNA on the gefitinib response of NSCLC cells A, Cell proliferation 24 h following siRNA treatment, in combination with gefitinib (0.1μM, PC-9; 1μM, A549), and left to grow for 96 h before proliferation rate was measured using resazurin. *P<0.05, **P<0.01, compared with gefitinib treated non-targeting control (NT). B, Apoptosis assay 54 h after siRNA treatment, in combination with gefitinib (0.1μM, PC-9; 1μM, A549) for 18 h, *P<0.05 and **P<0.01, compared with gefitinib treated NT control. Data represent mean apoptotic levels ± SEM (n=3).

Total AKT inhibition by siRNA significantly decreased growth of PC-9 cells to a similar extent as MK2206 ≥1μM (Fig. 1A). Gefitinib significantly decreased PC-9 cell proliferation when combined with AKT1 siRNA (p=0.013) and AKT2 siRNA (p<0.01), but not AKT3 siRNA (p=0.12), compared with gefitinib treated non-targeting control (NT) (Fig. 3A). Combination with gefitinib also led to an increase in PC-9 apoptosis levels. Although inhibition of total AKT induced the greatest amount of apoptosis (20.2% ± 2.12%) in combination with gefitinib, it wasn’t significantly more than that induced by AKT2 siRNA. In addition, AKT2 was the only isoform whose inhibition in the presence of gefitinib significantly increased the levels of apoptosis in PC-9 cells, compared with gefitinib treated NT siRNA (p<0.01) (Fig. 3B).

Although inhibiting total AKT levels had a significant effect on the growth of A549 cells in the presence of gefitinib, the results suggest that there is no specific isoform responsible for this effect. Furthermore, there is a suggestion that AKT1 signaling may be involved in resistance to gefitinib induced apoptosis of A549 cells (Fig. 3A and B).

### Isoform selective AKT inhibitors sensitise EGFR M+ cells to gefitinib

**Figure 4:**
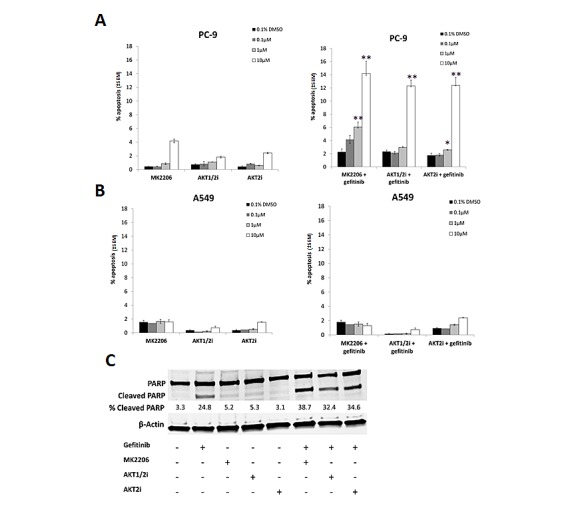
The effect of selective AKT inhibitors on the gefitinib induced apoptosis in NSCLC cells A, PC-9 and B, A549 cells were treated with 0-10μM of each of the AKT inhibitors (MK2206, AKT1/2i, and AKT2i) with or without 0.1/1μM of gefitinib for 18 h, *P<0.05, ** P<0.01, compared with either drug alone. C, PC-9 cells were treated with 10μM of each of the AKT inhibitors either alone or in combination with 0.1μM gefitinib before western blotting for PARP. Percentage of cleaved PARP was calculated by normalising to β-Actin levels and dividing the densitometry values of cleaved PARP by total PARP, + indicates the presence of the compound and – represents its absence. Blot is representative of at least 2 independent repeats.

In order to test whether more selective AKT inhibitors would have a similar effect to MK2206 in combination with gefitinib, cells were treated with 0-10μM of MK2206, isoform selective inhibitors of AKT 1 and 2 (AKT1/2i) [26], or AKT2 (AKT2i) [27], in combination with gefitinib (0.1μM/1μM) for 18 h, and apoptosis levels assessed using the IN Cell Analyzer. Each inhibitor alone induced only low levels of apoptosis in both PC-9 and A549 cells (Fig. 4A and B).

However, when each of the AKT inhibitors (10μM) was combined with gefitinib, there was a marked increase in apoptosis levels (MK-2006; 14.2% ± 1.85%, AKT1/2i; 12.3% ± 1.2%, and AKT2i; 12.4% ± 0.89%, p<0.01 for each dual treatment compared with either monotherapy alone) in PC-9 cells (Fig. 4A). With MK2206 and AKT2i, this combination also proved effective at the lower concentration of 1μM. A similar sensitivity to all three AKT inhibitors in combination with gefitinib was seen in the HCC-827 cells (Supplementary Fig. S2A). In H1975 cells however, only the combination of gefitinib and MK2206 proved to be effective (Supplementary Fig. S2B), while A549 cells were insensitive to all treatment (Fig. 4B).

In addition, following 24 h treatment with gefitinib and the AKT inhibitors, western blotting for PARP cleavage was carried out on PC-9 cells. Similarly to the IN Cell Analyzer results, while gefitinib monotherapy induced PARP cleavage, each of the inhibitors alone did not (Fig. 4C). However, when the AKT inhibitors (10μM) were combined with gefitinib, levels of cleaved PARP were significantly enhanced.

Proliferation assays revealed that the combination of AKT1/2i with gefitinib synergistically inhibited the growth of all cell lines tested, notably that of PC-9 and A549 (Supplementary table S1). The combination of gefitinib and AKT2i, synergistically inhibited the growth of HCC-827 cells but only had an additive effect on the other cell lines, as measured by CI (Supplementary table S2).

However, AKT1/2i and AKT2i have not been well characterised and therefore potential off target effects cannot be ruled out. In addition, as no AKT3 selective inhibitors are currently available, it is difficult to determine whether it too has a more pertinent role. Together with the siRNA data, this suggests that the AKT2 isoform is most important in the gefitinib induced response of EGFR M+ NSCLC cells.

### AKT inhibition induces autophagy

Treatment with AKT inhibitors induced puncta characteristic of autophagy as previously observed in other cancer cell types [17, 18]. After 24 h treatment with each of the AKT inhibitors (0-10μM), western blotting and IF staining was carried out for LC3 in PC-9 cells. LC3-I is recruited during autophagosome formation where it is cleaved and lipidated to form LC3-II, which migrates faster on a SDS-PAGE gel, and appears in autophagosome puncta when carrying out IF staining, serving as a characteristic trait of autophagic activation [28].

**Figure 5:**
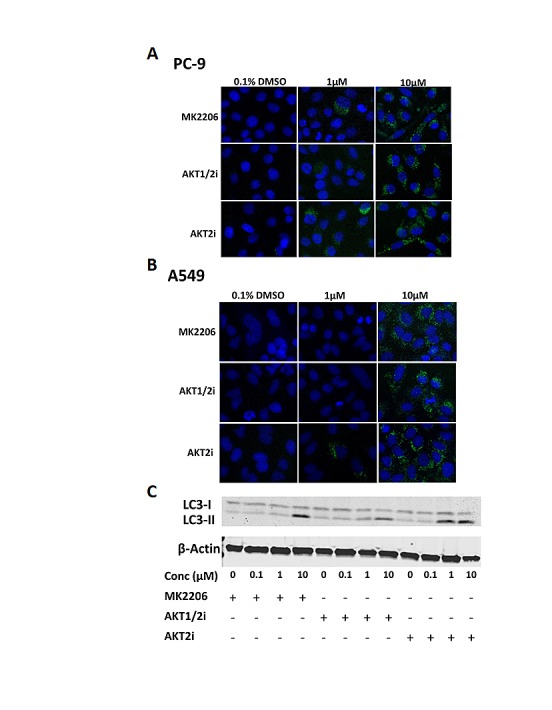
The effect of AKT inhibition on autophagy in NSCLC cells LC3 immunofluorescence of A, PC-9 and B, A549 cells treated with 0-10μM of each of the AKT inhibitors for 24 h (n=3). C, LC3 western blotting of PC-9 cells treated with 0-10μM of each of the AKT inhibitors for 24 h, + indicates the presence of the compound and – represents its absence. Blot representative of at least 2 independent repeats.

IF staining for LC3 revealed that the AKT inhibitors induced more autophagosome formation than in the untreated control, as shown by the presence of LC3 puncta. This effect was evident in each of the cell lines used, irrespective of their mutation status (Fig. 5A and, and Supplementary Fig. S3). In addition, western blotting demonstrated that the AKT inhibitors induced the conversion of LC3-I to LC3-II in a dose-dependent manner, with the effect being particularly evident at the highest dose of 10μM (Fig. 5C)

### Inhibiting autophagy with chloroquine sensitises EGFR M+ cells to combined treatment with EGFR and AKT inhibitors

In order to determine whether AKT inhibitor-induced autophagy is acting as a pro- or anti-survival mechanism in NSCLC cells, we indirectly blocked autophagy with a pharmacologic inhibitor chloroquine, which disturbs lysosome function. Briefly, the cells were treated with 10μM MK2206, AKT1/2i, or AKT2i, 0.1/1μM of gefitinib, and 20μM chloroquine for 18 h, before apoptosis was analyzed using the IN Cell Analyzer, and western blotting for LC3 and PARP cleavage.

**Figure 6:**
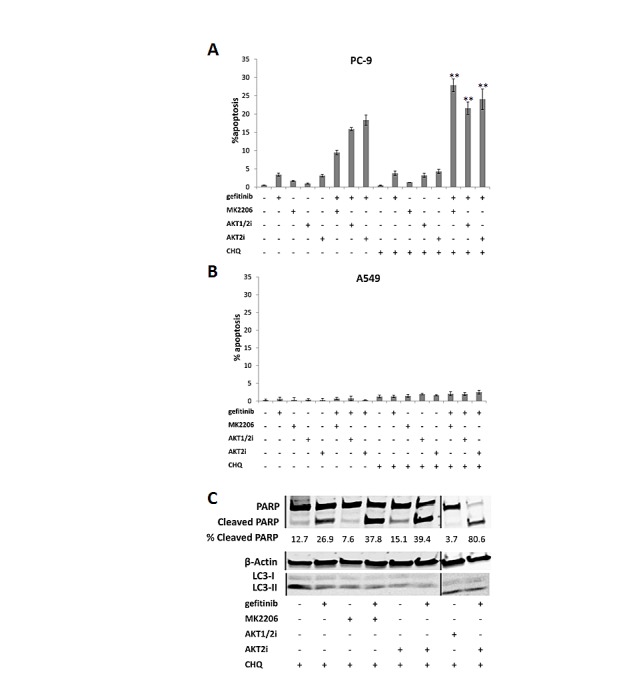
The effect of inhibiting autophagy on the gefitinib response of NSCLC cells Apoptosis assay of A, PC-9 and B, A549 cells treated with each of the AKT inhibitors (10μM), gefitinib (0.1/1μM), chloroquine (20μM), a combination of chloroquine with each of the drugs, or a combination of chloroquine, an AKT inhibitor, and gefitinib, for 18 h, **P<0.01 and *P<0.05, compared with gefitinib combined with corresponding AKT inhibitor. For presentation purposes, chloroquine is shortened to CHQ. Data represent mean apoptotic levels ± SEM (n=3). C, Western blotting for PARP cleavage in PC-9 cells treated with each of the AKT inhibitors (10μM) and chloroquine (20μM), with or without gefitinib (0.1μM) for 24 h, + indicates the presence of the compound and – represents its absence. Lines represent where the gel was cut and spliced to form this image. Blot is representative of at least 2 independent repeats.

While chloroquine induced very little apoptosis alone or in combination with the AKT inhibitors, when combined with gefitinib, apoptosis levels were increased in PC-9 cells (Fig. 6A). Notably, when chloroquine, gefitinib, and an AKT inhibitor were all combined, apoptosis was further enhanced. A similar result was observed in the HCC-827 cells (Supplementary Fig. S4A), but not in the A549 (Fig. 6B) or H1975 cells (Supplementary Fig. S4B).

These results were confirmed by western blotting where combining MK2206, AKT1/2i, or AKT2i, with gefitinib and chloroquine, increased the levels of cleaved PARP, compared with chloroquine and gefitinib dual therapy (Fig. 6C and Supplementary Fig. S5A). Chloroquine treatment was associated with an increased conversion of LC3-I to LC3-II, probably due to autophagosome accumulation, confirming that autophagy was successfully inhibited (Supplementary Fig. S5B) [29].

### Inhibiting autophagy with chloroquine further sensitises EGFR M+ cells to EGFR and AKT inhibition *in vivo*

Following our *in vitro* results, we wanted to test the efficiency of these drug combinations *in vivo*. HCC-827 cells were subcutaneously injected onto the flanks of Balb/C female nude mice (n=36). Once the tumors reached 100mm^3^ in volume, the mice were treated with vehicle control (p.o, days 1-3), gefitinib (p.o. 25mg/kg, days 1-3), MK2206 (p.o. 50mg/kg, days 1-3), chloroquine (i.p. 60mg/kg, days 1-3), gefitinib and chloroquine, MK2206 and chloroquine, gefitinib and MK2206, or gefitinib, MK2206, and chloroquine.

**Figure 7:**
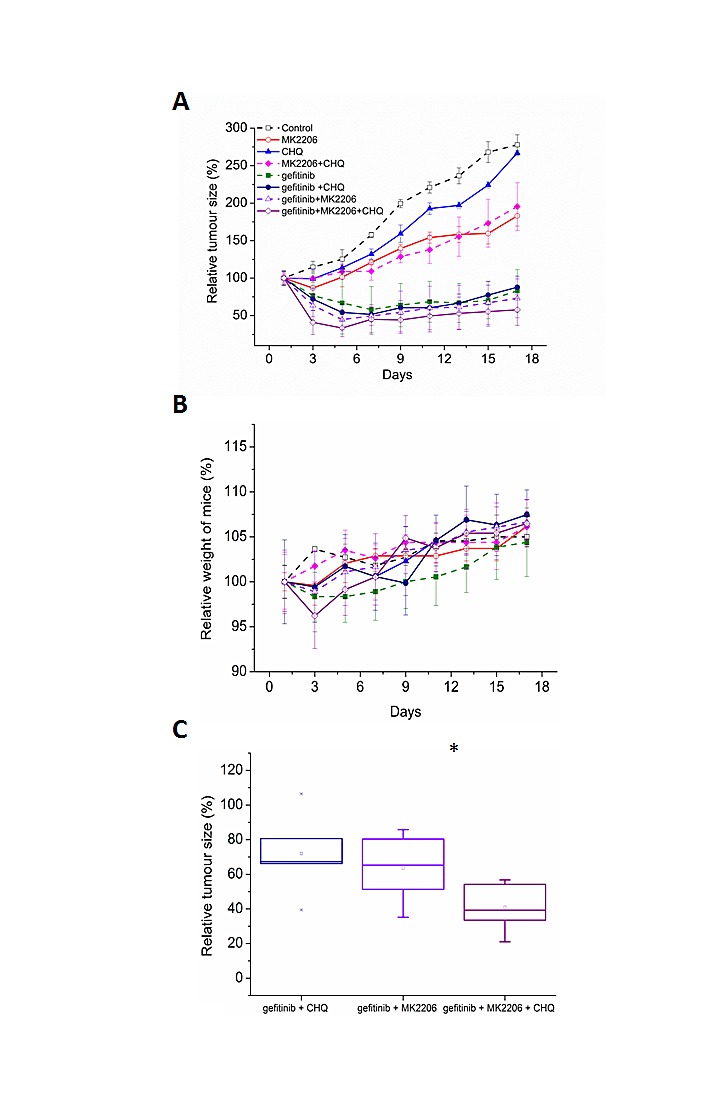
A, Balb/C nude mice harboring HCC-827 xenografts (100mm) were treated with vehicle control (p.o, days 1-3), gefitinib (p.o. 25mg/kg, days 1-3), MK2206 (p.o. 50mg/kg, days 1-3), chloroquine (i.p. 60mg/kg, days 1-3), gefitinib and chloroquine, MK2206 and chloroquine, gefitinib and MK2206, or gefitinib, MK2206, and chloroquine. Data are represented as relative tumor size normalised to day 0 (100%). B, relative weight of mice over course of experiment was calculated by normalising to day 0 (100%). C, relative tumor size of combination treatments on day 3 (immediately after treatment), *p<0.05.

Due to the acute response that we observed in proliferation and apoptosis assays, we investigated a 3 day dosing period *in vivo*. All treatments significantly inhibited tumor growth compared with the control (P<0.05, ANOVA), with the gefitinib containing regimens being the most effective (Fig. 7A). Overall, the treatments were well tolerated with no significant adverse clinical signs. Mice treated with the triple combination lost more weight than the other treatment groups, but weight loss was never above 4%, and the animals regained their weight a few days following treatment (Fig. 7B).

Adding MK2206 or chloroquine to gefitinib increased anti-tumor effects, but this did not reach statistical significance (Fig. 7A). Notably, the triple combination of adding chloroquine to gefitinib and MK2206 was more effective than either double combination, with tumors 35% smaller than gefitinib plus MK2206 alone (Fig. 7C). The benefit of the triple combination was only apparent at the end of the treatment period (Fig. 7A,), and was lost as tumors regrew suggesting sustained benefit would require prolonged dosing.

## DISCUSSION

Improving treatment outcomes for NSCLC patients with EGFR mutations remains an area of high unmet clinical need [3-5]. One common mechanism of resistance to EGFR inhibitors is overexpression and activation of EGFR downstream effectors including MAPK or PI3K/AKT [30], suggesting that one potential approach to improve efficacy is to combine EGFR TKIs with inhibitors of these other pathways. AKT activation has been reported in 51% of NSCLC patient samples and 74% of NSCLC cell lines, suggesting that combining EGFR and AKT inhibition may increase anti-tumor activity and prevent the occurrence of resistance to EGFR TKIs [31, 32]. Currently, the most advanced AKT inhibitor in clinical development is MK2206, an orally active, non-ATP competitive, allosteric pan-AKT inhibitor [16].

An approach we use in our laboratory is to identify agents that selectively induce rapid (≤24 h) apoptotic cell death in the presence of gefitinib, but only in EGFR M+ NSCLC cells. As part of this search we evaluated MK2206. Although this combination was synergistic in both EGFR M+ and EGFR WT cells in proliferation assays, the effects were modest and only apparent at lower doses. However, the effect on apoptosis was more marked and associated with significant reductions in clonogenic cell survival in EGFR M+ cells. In addition, the drug combination resulted in decreased pathway signaling (pEGFR, pAKT) in EGFR M+ cells compared with single drug treatments, which may in part explain the augmented levels of apoptosis seen in EGFR M+ cells treated with gefitinib and MK2206.

MK2206, as well as PI3K inhibitors, have been shown to act synergistically to improve anti-tumor activity in combination with EGFR TKIs in both the KRAS/EGFR WT and EGFR M+ NSCLC setting [30, 33, 34]. In one study, MK2206 in combination with erlotinib or lapatinib resulted in a synergistic inhibition of proliferation of both erlotinib sensitive and insensitive cells [35]. Combining MK2206 with gefitinib has also been shown to be successful in EGFR expressing malignant glioma cells, where combined therapy resulted in a synergistic increase in apoptosis and autophagy *in vitro*, and increased anti-tumor activity *in vivo* [36]. Due to this success, there are currently two on-going Phase I studies combining MK2206 with gefitinib in NSCLC patients (NCT01294306 and NCT01147211), one which is specifically enriched for EGFR mutations.

However, despite this relatively improved benefit of combining MK2206 and gefitinib in EGFR M+ cells, preclinical data using mouse models has shown that combined inhibition of both AKT1 and AKT2 can result in insulin resistance as well as hyperglycaemia and hyperinsulinaemia [37]. A dose-escalating phase I clinical trial of MK2206 demonstrated target inhibition in biomarker samples at plasma drug levels of greater than 50-65 nM which can be sustained at the maximum tolerated dose (60 mg QOD) [38]. However, adverse events including skin rash and hyperglycaemia [16], suggest that therapeutic benefit of pan-AKT inhibition may be limited, and that inhibiting all three AKT isoforms may not be the best approach to maximise clinical benefit.

Therefore, we investigated whether a specific AKT isoform is more important in regulating the effects of gefitinib in EGFR M+ cells. We initially attempted this with the use of AKT isoform selective siRNAs, and went on to validate our observations using isoform selective inhibitors of AKT 1 and 2, and AKT2. This data shows that inhibiting AKT2 with siRNA results in significantly increased sensitivity to both the anti-proliferative and apoptotic effects of gefitinib, with AKT1 also proving important in growth inhibition. AKT3 inhibition meanwhile did not have any significant effects. These effects were selective for EGFR M+ NSCLC cells (compared with EGFR WT), indicating that AKT2 and possibly AKT1, play an important role in conferring resistance of EGFR M+ cells to gefitinib induced apoptosis and growth inhibition.

The role of AKT2 in lung tumorigenesis remains unclear and studies have not yielded wholly consistent results. Using mouse Kras-dependent lung tumor models, AKT2 loss decreased lung tumor formation in the 4-(methylnitrosamine)-1-(3-pyridyl)-1-butanone (NNK) model, had no effect on a Kras(LA2) model, and increased tumor formation in a urethane-induced model [39]. In contrast, AKT1 was most important for tumor initiation and progression in these mouse lung tumor models [12]. The reason for this disparity may be due to this particular lung tumor model being induced by KRAS mutations, whereas the EGFR M+ cell lines used in our study are wild-type for KRAS. Furthermore, our data suggest that in A549 cells, which are KRAS mutant [40], AKT1 may be more important for determining EGFR TKI sensitivity. Additionally, AKT3, but not AKT2 depletion, was found to inhibit proliferation and survival of lung cancer derived disseminated human tumor cells [41].

Apart from apoptosis, AKT inhibition has also been shown to induce autophagy. For example, the pan-AKT inhibitor AZD5363 has recently been reported to induce autophagy in prostate cancer cells, by down-regulating the mTOR pathway [17]. Furthermore, prolonged down-regulation of AKT2 using siRNA induces conversion of LC3-I to LC3-II, resulting in cell death by autophagy of the mitochondria in breast cancer cell line MDA-MB231 [18]. Our data show that the selective AKT2i induces autophagy, though we cannot rule out any involvement of the other AKT isoforms. In addition, in our studies siRNA against total AKT did not induce autophagy (data not shown), consistent with a recent report from another group using A549 cells [19].

Autophagy has been shown to provide cancer cells with an energy source in order to help them survive in environments unfavorable for normal cells, suggesting that inhibiting autophagy may potentiate the effects of targeted therapies [42]. For example, it has been shown that inhibiting autophagy in HER2 overexpressing breast cancer cells, sensitised them to EGFR TKIs [43]. In addition, a more recent study has shown that autophagy inhibition by chloroquine further sensitises EGFR M+ NSCLC cells to erlotinib [44]. This is in accordance with our data, where the combination of gefitinib and chloroquine enhanced PARP cleavage by western blotting, compared with either treatment alone.

This is in contrast to a recent study, which has shown that inhibiting autophagy promotes tumor survival, and antagonises the effects of erlotinib in HCC-827 cells both *in vitro* and *in vivo*. In addition, the authors concluded that patients receiving EGFR TKIs should not be treated with autophagy inhibitors, as this might worsen rather than improve their prognosis [45]. Our data however, shows no evidence for antagonism of chloroquine and gefitinib in EGFR M+ cells *in vitro* or *in vivo.* Furthermore, when chloroquine was added to the combination of MK2206 and gefitinib in HCC-827 cells, it significantly increased apoptosis *in vitro* and decreased tumor growth of xenografts, suggesting that autophagy may provide a survival mechanism in the context of AKT inhibition.

Chloroquine is known to have pleiotropic effects. In addition to inhibiting autophagy, it has been reported to induce apoptosis at concentrations higher than 50μM [46, 47]. In this study we used 20μM chloroquine, which did not induce apoptosis but did affect autophagy. Chloroquine has been shown to sensitise prostate cancer cells with a PTEN deletion to AKT inhibition by AZD5363 [17, 48]. In contrast, when we combined MK2206 and chloroquine in EGFR M+ NSCLC cells, this did not have any added benefit, but these cells are highly dependent on EGFR rather than AKT signaling for survival.

In conclusion, this study implicates AKT2 signaling as a determinant of gefitinib resistance in EGFR M+ cells. Our data suggest combining AKT2 selective inhibitors with an EGFR TKI as an interesting matching target therapeutic approach in EGFR M+ NSCLC tumors [49]. Furthermore, our data suggest that the effect of this novel combination may be limited by a prosurvival autophagy response, and that combining chloroquine with EGFR and AKT inhibition in the EGFR M+ NSCLC patient subgroup may be of additional benefit.

## MATERIALS AND METHODS

### Cell lines and culture

HCC-827 (EGFR exon 19 mutation, DelE746_A750), H1975 (EGFR_L858R, EGFR_T790M), A549, (EGFR wild type (WT), KRAS G12S mutation), were acquired from the American Type Culture Collection (ATCC, Manassas, VA) in 2010-2012. PC-9 (EGFR exon 19 mutation, DelE746_A750) was kindly provided by Dr. Kazuto Nishio, Kinki University, Osaka, Japan in 04/2010. Upon receipt, all cell lines were authenticated by mitochondrial DNA sequencing and were passaged for no longer than 3 months post authentication [50]. Cell lines were routinely maintained in Advanced DMEM/F-12 (Invitrogen Inc., Paisley, UK) supplemented with 5% FBS (Invitrogen Inc.), GlutaMAX™, and Penicillin-Streptomycin (Sigma-Aldrich, Inc., Poole, Dorset, UK). Cells were cultured at 37°C in a humidified environment containing 7.5% CO_2_.

### Compounds

Gefitinib and MK2206 were obtained from Selleck Chemicals (Texas, USA) and stored as 10 mM stock solutions in DMSO at −20°C. The AKT Inhibitor VIII, Isozyme-selective Akti-1/2, and the AKT inhibitor XII isozyme selective, Akti-2 were purchased from Calbiochem (Darmstadt, Germany). Chloroquine was purchased from Sigma-Aldrich.

### *In vitro* cell proliferation assay

Cells (1 × 10^3^ in 100μl DMEM), were seeded into 96-well cell culture plates (Corning, Appleton Woods Limited, Birmingham, UK), and left overnight. Cells were treated in triplicate with gefitinib (0-0.4 μM for PC-9, and 0-8 μM for A549) or MK2206 (0-8 μM), either alone or in combination. Following 5 days incubation, resazurin (12.5μg/ml; 1:10 of total volume, Sigma-Aldrich) was added, and cell viability assessed by fluorescence intensity (POLARstar Omega; BMG Labtech GmbH, Germany) at 540 nm excitation and 590 nm emission. Data was expressed relative to DMSO treated controls.

In order to assess the effect of AKT siRNA on growth inhibition, 24h after transfection, cells were treated with 0.1% DMSO or gefitinib (0.1 μM), and left to grow for 5 days before growth rate was assessed using resazurin.

### IC50 determination

IC50 values were determined using CalcuSyn software (BIOSOFT®, Cambridge, UK), taking into account the average of all experiments.

### Combination index

In order to measure any potential synergistic drug interactions, combination studies were performed according to the median-effect method of Chou and Talalay [25, 51, 52]. Each drug alone was tested at concentrations of 8x, 4x, 2x, 1x, 0.5x, 0.25x, and 0.125x its IC50. For the combination, the fixed ratio (1:1) of each drug at 8x, 4x, 2x, 1x, 0.5x, 0.25x, and 0.125x its IC50 value was tested. Drug exposure was simultaneous. The data was analyzed using CalcuSyn software (BIOSOFT®).

### Clonogenic survival assay

Sensitivity to the combination of gefitinib and MK2206 was assessed using clonogenic survival assays. Cells were seeded in 6-well culture plates (Appleton Woods Ltd.), allowed to settle overnight, and treated in duplicate with gefitinib (0.1/1 μM), MK2206 (1 μM), or a combination of the two, for 24 h. Cells were washed, and incubated to allow for colony formation. Colonies were fixed and stained in crystal violet (0.5% crystal violet, 4.5% acetic acid, 20% ddH_2_O, 75% MeOH), and colonies (≥50 cells) counted.

### SDS-PAGE and western blot analysis

Cells were treated with gefitinib (0.1 μM) and/or MK2206 (1 μM), or 0.1% DMSO (control) for 24 h. The cells were lysed on ice in lysis buffer (50mM Tris-Hcl pH7.6, 137mM NaCl, 10% glycerol, 0.1% Igepal, 0.1% SDS, 50mM NaF, protease inhibitor (Roche, Basel, Switzerland)), and 50μg protein per lane was loaded onto a 10% Mini-PROTEAN® Precast TGX Gel (Bio-Rad, Hemel Hempstead, UK). After electrophoresis, protein was transferred onto a nitrocellulose membrane (Bio-Rad), blocked in LI-COR blocking buffer (LI-COR Biosciences, Nebraska, USA), and incubated overnight with primary antibodies (supplementary table 3). IRDye® secondary antibodies (LI-COR Biosciences) were added, and bands detected using an Odyssey Infrared Imager (LI-COR Biosciences). Band quantification was performed using ImageJ. To quantify cleaved PARP, bands for both total and cleaved PARP were normalized to the corresponding β-actin levels.

### Quantification of apoptosis

Cells were plated into 96-well plates (1 × 10^5^ cells/well) in phenol red free DMEM/F-12 media (Invitrogen Inc.), and incubated overnight. The next day, cells were treated in triplicate with either gefitinib (0.1 μM/1 μM) and/or MK2206 (1 μM), and incubated for 18 h. Hoechst 33258 (25 μM) was added and incubation continued for 45-60 min.

The fluorescent cells were imaged (360 nm excitation, 460 nm emission) using the IN Cell Analyzer 1000 (GE Healthcare Life Sciences, Buckinghamshire, UK). Using automated software analysis (GE Healthcare Life Sciences), cells with condensed nuclei that have a high nuclear intensity are defined as apoptotic, and total number and percent apoptotic cells calculated.

In order to assess the effect of AKT siRNA on apoptosis, 54 h after transfection, cells were treated with 0.1% DMSO or gefitinib (0.1 μM) for 18 h before the addition of Hoechst. Apoptosis was analyzed as above.

### Transfection of AKT small interfering RNA (siRNA)

siGENOME SMARTpool® siRNA containing 3 specific siRNAs for AKT1 (M-003000-03), AKT2 (M-003001-02), AKT3 (M-003002-02), and non-targeting control (D-001206-13-05), were purchased from DHARMACON Research Inc. (Thermo Fisher Scientific, Lafayette, CO). SignalSilence AKT (I) siRNA (6211s) was purchased from Cell Signaling Technology (Beverly, MA).

Briefly, PC-9 and A549 cells were grown to 50% confluency, and then transfected with siRNA (25nM) using Lipofectamine™ 2000 (Invitrogen Inc.). Cells were harvested up to 72h later, and analyzed for the expression of each protein, using western blotting. Controls were transfected with non-targeting siRNA, and grown under similar conditions. Knock down levels were determined by band quantification using Image J.

### Immunoflourescent staining of LC3

Cells were seeded into 96 well plates at a density of 10,000 cells per well and left to adhere overnight before treatment with the AKT inhibitors (0, 0.1, 1, and 10 μM) for 24 h. Cells were fixed in 4% paraformaldehyde and then permeabilised in 0.25% Triton-X in PBS. Cells were blocked in 3% BSA in PBS, treated overnight with an LCA3/B (G40) primary antibody (Cell Signaling Technology) at a concentration of 1:1000. This was followed by 1 h incubation with an Alexa555 conjugated secondary antibody (Invitrogen Inc.) in darkness, and 5 minutes with DNA stain DAPI (1mg/ml). LC3 staining was assessed using the IN Cell Analyzer 1000 (GE Healthcare Life Sciences).

### Response of NSCLC xenografts to gefitinib, MK2206 and chloroquine

Animal procedures were carried out after local ethical committee review under a project license issued by the UK Home Office under the UK Animals (Scientific Procedures) Act 1986, and performed according to national guidelines [53]. HCC-827 (0.1 ml, 5.0x10^6^) cells in 50% matrigel were subcutaneously injected into a single site on the back of an anaesthetized Balb/C female nude mice (CAnN.Cg-*Foxn1^nu^*/Crl, 6-8 weeks old, 15-18g, Charles River Laboratories International, Inc., n=36). When tumors reached an average size of 100 mm^3^, the mice were randomized to receive gefitinib (25 mg/kg p.o. in 1% polysorbate 20, days 1-3 (n=3)), MK2206 (60 mg/kg p.o. in 1% polysorbate 20, days 1-3 (n=3)), chloroquine (50mg/kg IP in water, days 1-3 (n=3)), or the following combinations; gefitinib and chloroquine (n=5), MK2206 and chloroquine (n=5), gefitinib and MK2206 (n=5), and gefitinib, MK2206, and chloroquine (n=5). Control animals received 1% polysorbate 20 p.o., days 1-3. For combination treatments, gefitinib and MK2206 were given simultaneously, while chloroquine was given 2 h before treatment with gefitinib or MK2206.

### Statistics

Statistics were calculated using Microsoft Excel 2011. T-Tests were carried out in order to determine statistical relevance (P<0.05). The *in vivo* data was analyzed using ANOVA (SPSS), with treatment group and days after treatment used as factors.

## SUPPLEMENTARY MATERIAL FIGURES AND TABLES


