# Supplementary material for: Combining AKT inhibition with chloroquine and gefitinib prevents compensatory autophagy and induces cell death in EGFR mutated NSCLC cells
Source: Oncotarget. 2014 May 27;5(13):4765–78. doi: 10.18632/oncotarget.2017 (PMC4148097; doi:10.18632/oncotarget.2017)
Supplement: Supplementary file 1 [file oncotarget-05-4765-s001.pdf]

Combining AKT inhibition with chloroquine and gefitinib prevents compensatory autophagy and induces cell death in EGFR mutated NSCLC cells

Supplementary Material

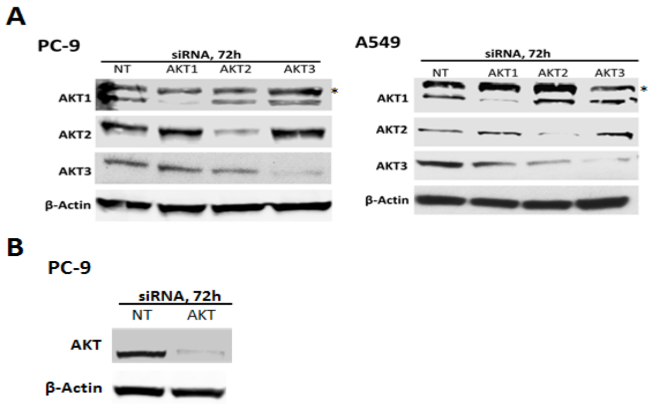

Figure S1: Inhibition of AKT isoforms using siRNA. A. PC-9 and A549 were treated with siRNA (25nm, siGENOME SMARTpool®) against each AKT isoform, and effects verified by western blotting 72 h later. The top band of AKT1, marked with an * is a non-specific band and hence knock-down is determined by loss of the bottom band. B. PC-9 and A549 were treated with 2 specific siRNAs (50nM, SignalSilence AKT), and effects verified by western blotting 72 h later.

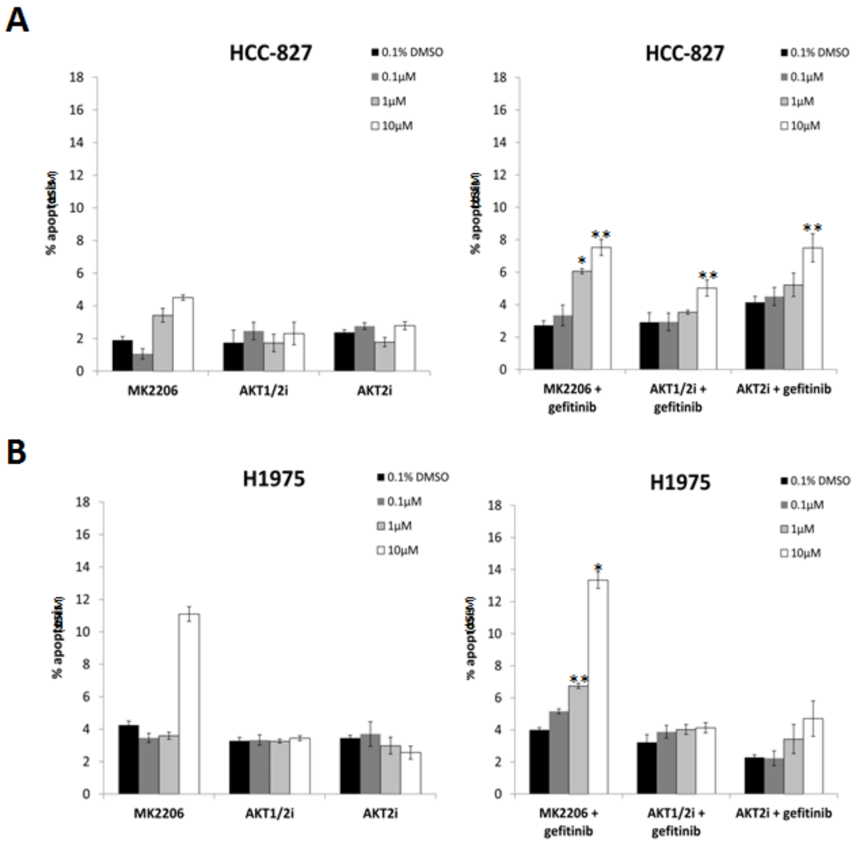

Figure S2: The effect of selective AKT inhibitors on the gefitinib induced apoptosis in NSCLC cells. Apoptosis assay of A, HCC827 and B, H1975 cells treated with 0-10µM of each of the AKT inhibitors (MK2206, AKT1/2i, and AKT2i), with or without 0.1/1µM of gefitinib for 18 h, *P<0.05, ** P<0.01, compared with either drug alone. Data represents mean apoptotic levels ± SEM (n=3).

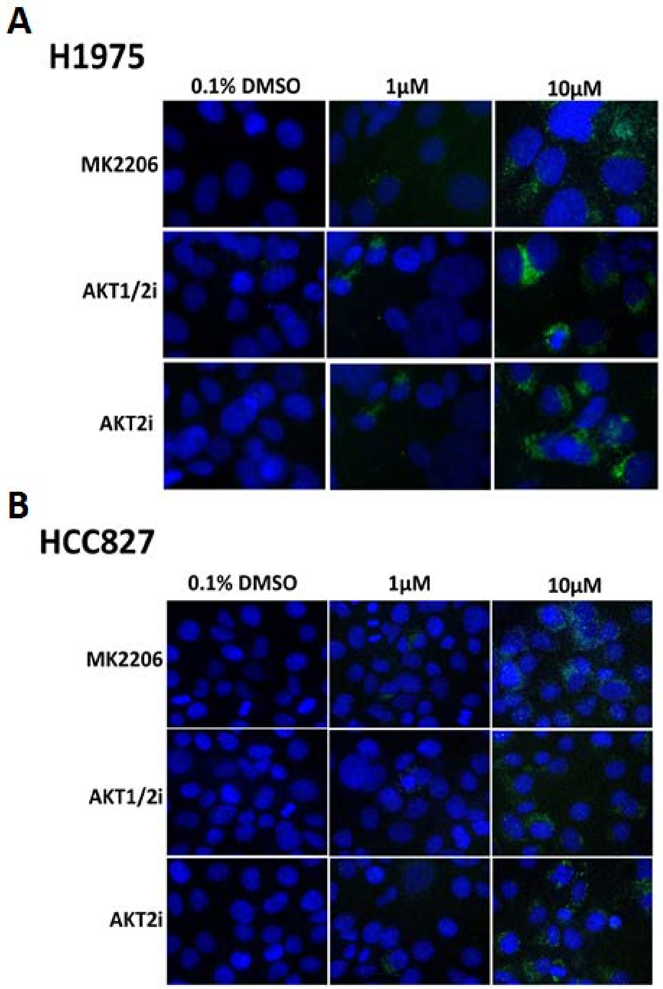

Figure S3: The effect of AKT inhibition on autophagy in NSCLC cells. LC3 immunoflourescence of A, HCC827 and B, H1975 cells treated with 0-10μM of each of the AKT inhibitors for 24 h (n=3).

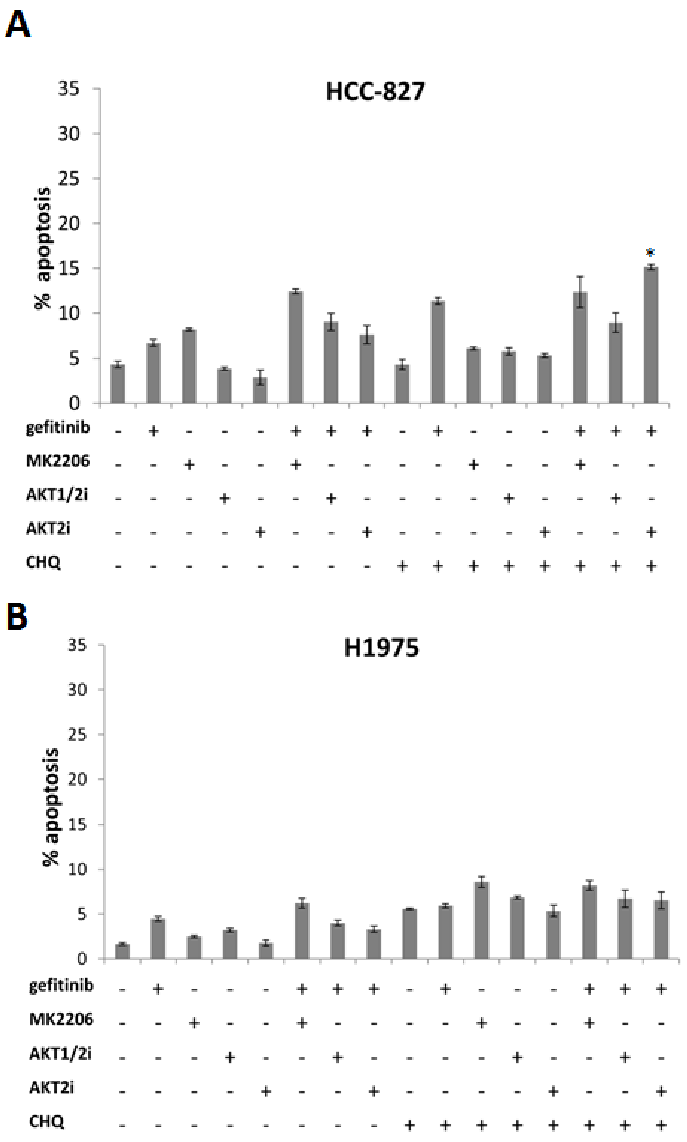

Figure S4: The effect of inhibiting autophagy on the gefitinib response of NSCLC cells. A, HCC-827 and B, H1975 cells were treated with each of the AKT inhibitors (10µM), gefitinib (0.1/1µM), Chloroquine (20µM), a combination of Chloroquine with each of the drugs, or a combination of Chloroquine, an AKT inhibitor, and gefitinib, for 18 h. The cells were stained with Hoechst and imaged using the IN Cell analyzer, *$p<0.05$ compared to combination of gefitinib and chloroquine, as well as AKT2i and gefitinib, + indicates the presence of the compound and – represents its absence. Data represent mean apoptosis levels ± SEM (n=3).

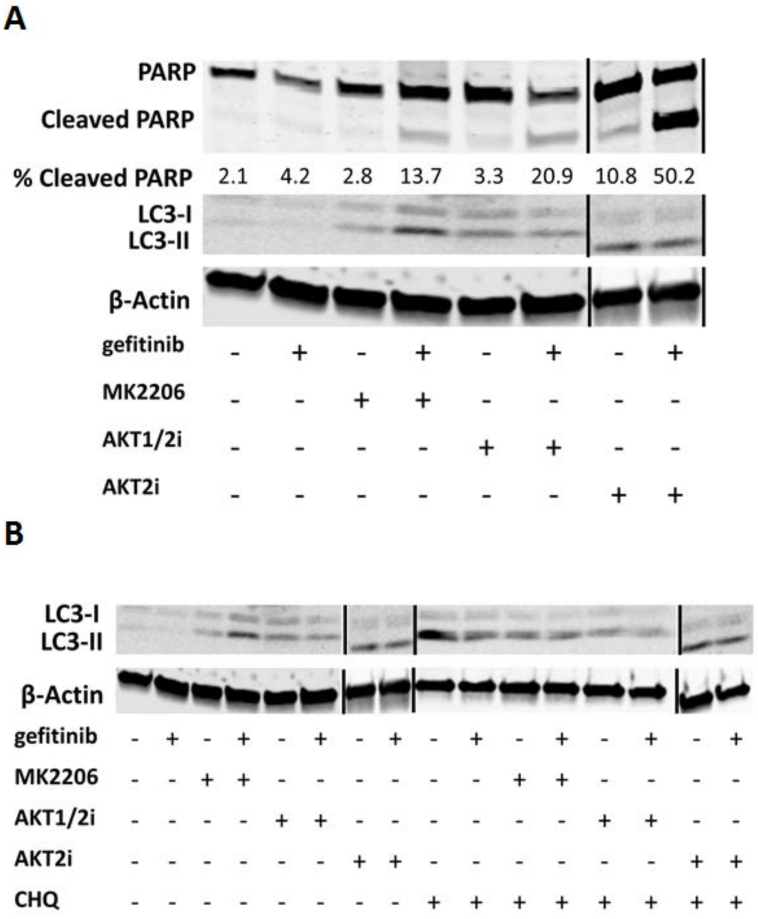

Figure S5: Remainder of the blot in figure 6c. A, PC-9 cells were treated with each of the AKT inhibitors (10µM) with or without gefitinib (0.1µM) for 24 h before the cells were lysed and western blotting carried out for PARP cleavage. B, the same samples used in fig. 6c and S5A were analysed for LC3B levels using western blotting, + indicates the presence of the compound and – represents its absence. Lines represent where the gel was cut and spliced to form this image.

Table S1: Combination index values calculated using Calcusyn for the combination of AKT1/2 inhibitor and gefitinib in NSCLC cells

Cell line	Combination index (±SEM)		
	ED50	ED75	ED90
PC-9	0.25±0.02	0.45±0.06	0.83±0.12
HCC-827	0.41±0.18	0.67±0.25	1.90±0.74
A549	0.14±0.10	0.11±0.07	0.10±0.05
H1975	1.13±0.29	0.66±0.18	0.52±0.30

Table S2: Combination index values calculated using Calcusyn for the combination of AKT2 inhibitor and gefitinib in NSCLC cells

Cell line	Combination index (±SEM)		
	ED50	ED75	ED90
PC-9	0.99±0.12	0.94±0.07	0.96±0.06
HCC-827	0.19±0.11	0.22±0.02	0.34±0.13
A549	0.95±0.49	0.87±0.64	1.83±0.72
H1975	0.86±0.53	0.89±0.52	0.97±0.48

Table S3: Antibodies used in this study.

Antibody	Species	Dilution	Manufacturer
EGFR	Rabbit	1:2000	Cell signaling technologies, Inc. (Danvers, USA)
Phospho-EGFR (Y1068)	Rabbit	1:2000	Invitrogen, Inc. (Paisley, UK)
AKT	Rabbit	1:2000	Cell signaling technologies, Inc. (Danvers, USA)
Phospo-AKT (Ser473)	Rabbit	1:1000	Cell signaling technologies, Inc. (Danvers, USA)
AKT1 (2H10)	Mouse	1:1000	Cell signaling technologies, Inc. (Danvers, USA)
AKT2 (D6G4)	Rabbit	1:1000	Cell signaling technologies, Inc. (Danvers, USA)
AKT3 (L47B1)	Mouse	1:1000	Cell signaling technologies, Inc. (Danvers, USA)
S6 (S4D2)	Mouse	1:1000	Cell signaling technologies, Inc. (Danvers, USA)
Phospho-S6 (S240/244)	Rabbit	1:1000	Cell signaling technologies, Inc. (Danvers, USA)
PARP	Rabbit	1:2000	Cell signaling technologies, Inc. (Danvers, USA)
LCA3/B	Rabbit	1:1000	Cell signaling technologies, Inc. (Danvers, USA)
ß-actin	Mouse	1:5000	Abcam (Cambridge, UK)
IRDye® 800CW goat anti-rabbit	Goat	1;15,000	LI-COR Biosciences (Nebraska, USA)
IRDye® 680LT goat anti-mouse	Goat	1:25,000	LI-COR Biosciences (Nebraska, USA)
Alexa Fluor®555 goat anti-rabbit	Goat	1:1500	Invitrogen, Inc. (Paisley, UK)
